# Hospital-Onset Bloodstream Infections Caused by Eight Sentinel Bacteria: A Nationwide Study in Israel, 2018–2019

**DOI:** 10.3390/microorganisms10051009

**Published:** 2022-05-11

**Authors:** Amir Nutman, Liat Wullfhart, Elizabeth Temkin, Sarah F. Feldman, Vered Schechner, Mitchell J. Schwaber, Yehuda Carmeli

**Affiliations:** 1 National Institute for Antibiotic Resistance and Infection Control, Ministry of Health, Tel-Aviv Sourasky Medical Center, Tel Aviv 6423906, Israel; liatw@tlvmc.gov.il (L.W.); lizt@tlvmc.gov.il (E.T.); sarahf@tlvmc.gov.il (S.F.F.); vereds@tlvmc.gov.il (V.S.); mitchells@tlvmc.gov.il (M.J.S.); yehudac@tlvmc.gov.il (Y.C.); 2 Sackler Faculty of Medicine, Tel Aviv University, Tel Aviv 6997801, Israel

**Keywords:** hospital-onset bloodstream infections, bacteremia surveillance, incidence, mortality, antimicrobial resistance, nationwide study

## Abstract

Nationwide studies on hospital-onset bloodstream infections (HO-BSIs) are scarce. To describe incidence, mortality and antimicrobial resistance (AMR) of HO-BSI caused by eight sentinel bacteria in Israel, we used laboratory-based BSI surveillance data from 1 January 2018 to 31 December 2019. All hospitals reported positive blood cultures growing *Escherichia coli*, *Klebsiella pneumoniae*, *Pseudomonas aeruginosa*, *Acinetobacter baumannii*, *Streptococcus pneumoniae*, *Staphylococcus aureus*, *Enterococcus faecalis* and *Enterococcus faecium*. We calculated HO-BSI incidence and 14-day, 30-day and 1-year mortality in adults. We performed multivariable logistic regression to identify predictors of 30-day mortality. The study included 6752 HO-BSI events: *K. pneumoniae* (1659, 22.1%), *E. coli* (1491, 19.8%), *S. aureus* (1315, 17.5%), *P. aeruginosa* (1175, 15.6%), *E. faecalis* (778, 10.4%), *A. baumannii* (654, 8.7%), *E. faecium* (405, 5.4%) and *S. pneumoniae* (43, 0.6%). Overall incidence was 2.84/1000 admissions (95% CI: 2.77–2.91) and 6.88/10,000 patient-days (95% CI: 6.72–7.05). AMR isolates accounted for 44.2% of events. Fourteen-day, thirty-day and one-year mortality were 30.6% (95% CI: 28.5%–32.8%), 40.2% (95% CI: 38.2%–42.1%) and 66.5% (95% CI: 64.7%–68.3%), respectively. Organisms with highest risk for 30-day mortality (compared with *E. coli*) were *A. baumannii* (OR 2.85; 95% CI: 2.3–3.55), *E. faecium* (OR 2.16; 95% CI: 1.66–2.79) and *S. pneumoniae* (OR 2.36; 95% CI: 1.21–4.59). Mortality was higher in AMR isolates (OR 1.57; 95% CI: 1.4–1.77). This study highlights the incidence, associated high mortality and important role of antibiotic resistance in HO-BSI.

## 1. Introduction

Hospital-onset bloodstream infections (HO-BSIs) have a substantial impact on patient morbidity, mortality, length of hospital stay and costs [1,2]. A point prevalence survey conducted by the European Center for Disease Prevention and Control (ECDC) in 2016–2017 estimated that 6.5% of hospitalized patients had at least one episode of a healthcare-associated infection (HAI). Of these, 10.8% were HO-BSIs, translating to an estimated annual incidence of 375,000 HO-BSIs in Europe [3]. Goto et al. estimated 102,000–120,000 HO-BSIs in the USA annually, with a case-fatality rate (CFR) of 15–30% [4].

BSI surveillance is important to determine the burden of disease, to identify trends in incidence and to detect emerging pathogens and emergence of antibiotic resistance [5]. Antibiotic resistance among the predominant nosocomial bacterial pathogens, including Gram-negative bacilli, *Staphylococcus aureus* and *Enterococci*, has risen substantially in the past few decades [6]; this trend has stabilized in Europe over the past few years [7]. Emerging multidrug-resistant organisms threaten the effectiveness of antimicrobial treatment. Inappropriate empirical antibiotic treatment has been associated with increased mortality from BSI [8].

Data on the incidence of HO-BSI can inform clinicians, public health officials, policymakers and the public. These data can be used to raise awareness, encourage the implementation of preventive measures, improve diagnosis and treatment and allocate resources. Most reports on the incidence and prevalence of HO-BSI are based on hospital surveillance studies, and population-based studies, specifically nationwide studies, are scarce [4]. Nationwide surveillance systems have the advantage of including a large number of hospitals and varied hospital types, as well as wide geographic catchment areas [4].

In Israel, the National Institute for Antibiotic Resistance and Infection Control (NIARIC) has monitored BSI caused by eight bacteria of major public health importance (sentinel bacteria) in all acute care hospitals since 2012. The aim of this study was to provide a national estimate of the incidence of HO-BSI caused by these sentinel bacteria, the associated short-term and long-term mortality and the impact of antimicrobial resistance (AMR).

## 2. Materials and Methods

We followed the STROBE guidelines for observational studies. Supplementary Table S1 lists the elements of the guidelines and where they are found in our study.

### 2.1. Study Participants

We used laboratory-based surveillance data reported to the NIARIC between 1 January 2018 and 31 December 2019. Data were provided by all 29 acute care hospitals in Israel, together accounting for 14,618 acute care hospital beds. We included all BSIs reported for hospitalized adult patients (age ≥ 18 years at onset of bacteremia). We excluded one children’s hospital and one small community hospital with no adult internal medicine ward or ICU.

### 2.2. Data Collection

Hospital laboratories submitted monthly mandatory reports on all blood cultures positive for the following sentinel bacteria: *Escherichia coli*, *Klebsiella pneumoniae*, *Pseudomonas aeruginosa*, *Acinetobacter baumannii*, *Streptococcus pneumoniae*, *Staphylococcus aureus*, *Enterococcus faecalis* and *Enterococcus faecium*; these are the same pathogens monitored by the European Antimicrobial Resistance Surveillance Network (EARS-Net) [7]. Submitted data included patients’ unique identity number (UID), sex, date of birth, admission date, specimen collection date, ward where the culture was taken, type of culture (aerobic, anaerobic or pediatric), isolated organism and results of antibiotic susceptibility testing (AST).

We used patients’ UIDs to search for dates of death in the death registry of the Ministry of the Interior. The death registry data were available through 30 April 2020. We obtained data on the number of admissions and patient-days in hospitals from the Israel Ministry of Health [9].

### 2.3. Laboratory Methods

Blood cultures were processed at the participating laboratories according to each institution’s routine methods for isolate identification and AST.

### 2.4. Definitions

A BSI event was defined as growth of a sentinel bacteria in blood culture. HO-BSI was defined as a BSI with onset on or after the 4th hospital day, as per CDC guidance for hospital-onset LabID events [10]. The date the first positive blood culture was taken was regarded as the onset of the BSI event. Another blood culture positive for the same organism within 14 days was considered the same event; after 14 days it was defined as a new event [10]. Department of HO-BSI onset was determined according to the patient ward at the time the culture was taken.

Antibiotic susceptibility was based on the first isolate that defined the BSI event, consistent with the method used by EARS-Net [7]. AMR was defined by resistance to key antimicrobial agents in accordance with the WHO priority pathogen list [11]: penicillin resistance in *S. pneumoniae*; oxacillin or cefoxitin resistance in *S. aureus*; vancomycin or teicoplanin resistance in *E. faecalis* and *E. faecium*; extended-spectrum cephalosporin (3rd or 4th generation) resistance in *E. coli* and *K. pneumoniae* and non-susceptibility to imipenem or meropenem in *E. coli*, *K. pneumoniae*, *A. baumannii* and *P. aeruginosa*.

### 2.5. Incidence

Incidence rates (IRs) were calculated as the number of HO-BSI events per 1000 admissions and per 10,000 patient-days. Because the average length of stay (LOS) in acute care hospitals in Israel is 4 days [9], most patients are not at risk for HO-BSI, and these denominators may not accurately fit the period at risk. Therefore, we also used as denominators the number of admissions with LOS longer than 3 days (i.e., admissions at risk) and the number of patient-days after day 3 (i.e., patient-days at risk).

### 2.6. Mortality

We calculated 14-day, 30-day and 1-year mortality. For this calculation, we excluded patients with a missing or invalid UID, as they could not be matched to the death registry. To calculate 1-year mortality, we included only patients with a potential follow-up period of at least 1 year up to the date we accessed the death registry, i.e., BSI onset prior to 30 April 2019. For patients with multiple BSI events meeting the definition of HO-BSI, each event was analyzed separately.

### 2.7. Statistical Analysis

Patient and HO-BSI event characteristics were expressed as mean and standard deviation (SD), median and interquartile range (IQR) or percentage, as appropriate. We computed IR with 95% confidence intervals (CIs) overall and stratified by organism, hospital size, hospital day of onset and AMR. We calculated 14-day, 30-day and 1-year mortality with 95% CI overall and stratified by organism and by risk factor categories including age, sex, hospital size, hospital day of onset, department, AMR and BSI events with at least one other sentinel organism isolated within 5 days. Comparisons of mortality between risk factor categories were performed using the chi-square test. To identify predictors of 30-day mortality, we constructed a multivariable logistic regression model. All risk factors were modeled as categorical, except for age, which was modeled as continuous. All variables with a *p*-value ≤ 0.05 in univariate analysis were included in the multivariable model. No stepwise procedure was further needed, as all variables that were significant in the univariate analysis remained significant or marginally significant in the multivariable model. Analyses were performed in SAS version 9.4 (SAS Institute, Cary, NC, USA).

### 2.8. Ethical Considerations

The study was approved by the jurisdictional institutional review board (IRB). The requirement for informed consent was waived for this analysis of routinely collected surveillance data.

## 3. Results

### 3.1. Study Participants

During the two-year study period there were 6752 HO-BSI events caused by sentinel bacteria: *K. pneumoniae* (1659, 22.1%), *E. coli* (1491, 19.8%), *S. aureus* (1315, 17.5%), *P. aeruginosa* (1175, 15.6%), *E. faecalis* (778, 10.4%), *A. baumannii* (654, 8.7%), *E. faecium* (405, 5.4%) and *S. pneumoniae* (43, 0.6%). In 10% of HO-BSI events, at least one other sentinel organism was isolated within 5 days. A description of HO-BSI events is presented in Table 1. The mean patient age was 68.5 (±16.3) years, and 42.6% of events occurred in females. HO-BSI occurred after a median of 15 days (IQR: 8–29) after admission. The majority (67.1%) of HO-BSI events occurred in medical wards, and AMR organisms were isolated in 44.2% of HO-BSI events.

### 3.2. Incidence

Data on HO-BSI incidence by sentinel bacteria and hospitalization characteristics are presented in Table 2. Overall, the IR for HO-BSI by sentinel bacteria was 2.84 per 1000 admissions (95% CI: 2.77–2.91) and 6.88 per 10,000 patient-days (95% CI: 6.72–7.05). When counting only hospitalizations at risk (i.e., with LOS > 3 days), the IR was 8.66 per 1000 admissions (95% CI: 8.45–8.86) and 9.63 per 10,000 patient-days (95% CI: 6.72–7.05). IR increased with hospital size, from 1.61 per 1000 admissions (95% CI: 1.49–1.73) in hospitals with less than 300 hospital beds to 3.43 per 1000 admissions (95% CI: 3.32–3.55) in hospitals with over 700 beds.

**Figure 1:**
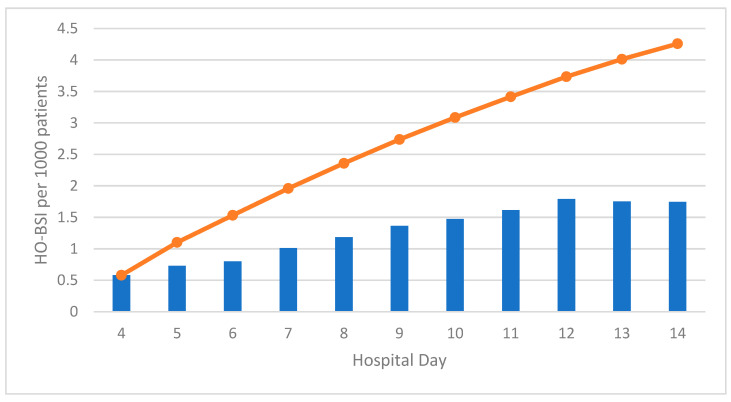
Incidence (bars) and cumulative incidence (line) of hospital-onset bloodstream infections (HO-BSIs) caused by sentinel bacteria, by hospital day of onset.

**Figure 2:**
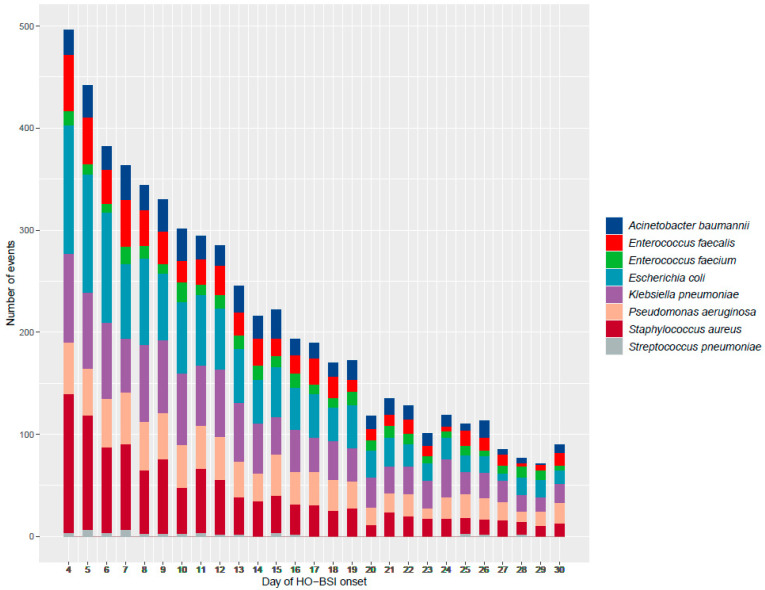
Number of sentinel bacteria hospital-onset bloodstream infections (HO-BSIs), by hospital day of onset and organism.

Figure 1 shows overall HO-BSI incidence for sentinel bacteria by day of onset. Incidence gradually tripled from 0.5 per 1000 patients hospitalized on day 4 to 1.5 per 1000 patients hospitalized on day 10 and stabilized at 1.75 per 1000 patients hospitalized on day 12 and onwards. The cumulative number of events caused by each sentinel bacteria by hospital day is presented in Figure 2.

**Figure 3:**
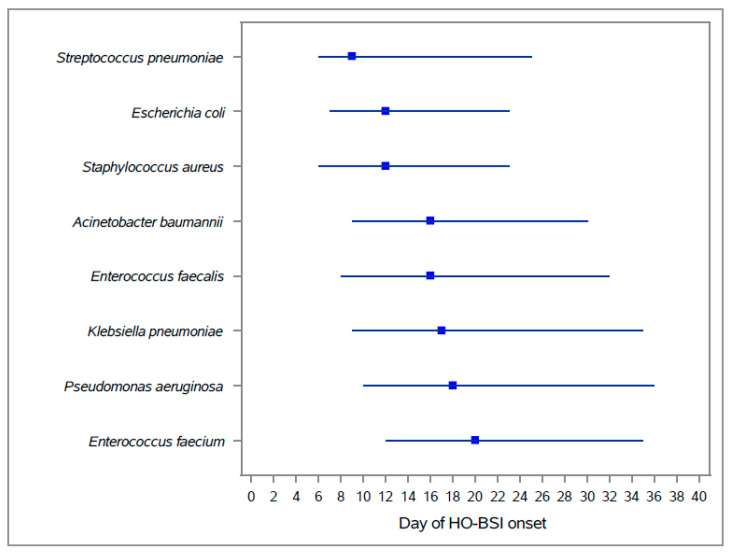
Days from admission to hospital-onset bloodstream infection (HO-BSI) onset, by organism. Square—median; whiskers—interquartile range.

The day of HO-BSI onset differed by organism (Figure 3): *E. coli*, *S. aureus* and *S. pneumoniae* were early HO-BSI events (median time from admission to infection 9–12 days), whereas *K. pneumoniae*, *P. aeruginosa*, *Enterococci* and *A. baumannii* were late BSI events (median time from admission to infection 16–20 days).

**Figure 4:**
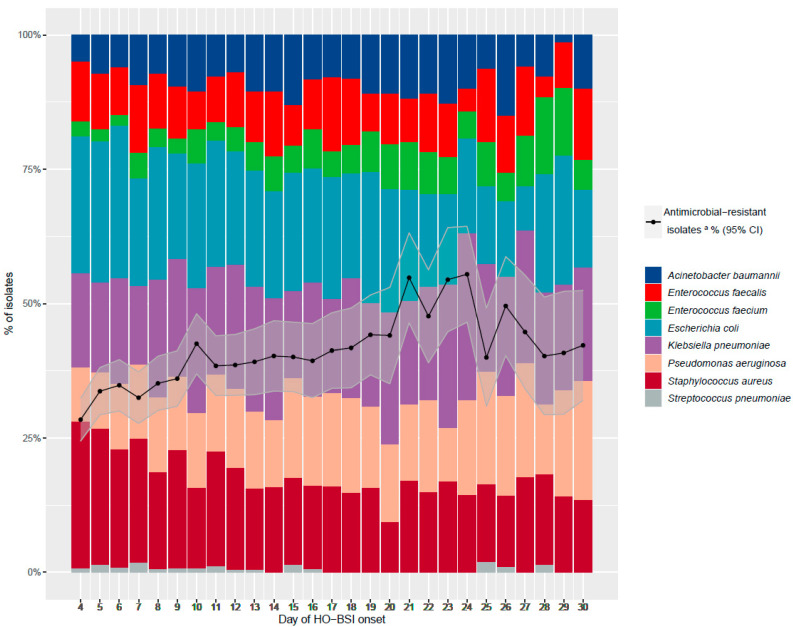
Distribution of sentinel organisms and percentage of antimicrobial-resistant isolates ^a^, by day of hospital-onset bloodstream infection (HO-BSI) onset. ^a^ Defined as resistance to key antimicrobial agents: penicillin resistance in *S. pneumoniae*; oxacillin or cefoxitin resistance in *S. aureus*; vancomycin or teicoplanin resistance in *E. faecalis* and *E. faecium*; extended-spectrum cephalosporin (third and fourth generation) resistance in *E. coli* and *K. pneumonia* and non-susceptibility to imipenem or meropenem in *E. coli*, *K. pneumoniae*, *A. baumannii* and *P. aeruginosa*.

The distribution of organisms according to the week of HO-BSI onset and proportion of AMR organisms is presented in Table 3. As the day of onset increased, *E. coli* and *S. aureus* accounted for a lower proportion of HO-BSI, and the proportion of HO-BSI caused by AMR organisms increased (Table 3 and Figure 4).

### 3.3. Mortality

Data on mortality are presented in Table 4. Survival data were available for 5907 (87.5%) events (845 events were excluded from this analysis due to invalid or missing UID), and 1-year survival data were available for 3839 (56.9%) events. The overall 14-day mortality was 30.6% (95% CI: 28.5–32.8%), 30-day mortality was 40.2% (95% CI: 38.2–42.1%) and 1-year mortality was 66.5% (95%: CI 64.7–68.3%). At all three time points, mortality was highest for *A. baumannii* BSI and lowest for *E. coli* BSI, and higher for HO-BSI caused by AMR organisms as compared to susceptible organisms. Short-term mortality (14-day and 30-day) did not vary significantly by hospital bed size; however, 1-year mortality was higher in hospitals with fewer than 300 beds as compared to larger hospitals.

Multivariable analysis of risk factors for death within 30 days among patients with HO-BSI is presented in Table 4. In univariate analysis, hospital size was not significantly associated with 30-day mortality and therefore was excluded from the multivariable model. The highest risk organisms (as compared to *E. coli*) were *A. baumannii* (adjusted OR (aOR) 2.85; 95% CI: 2.3–3.55), *E. faecium* (aOR 2.16; 95% CI 1.66–2.79) and *S. pneumoniae* (aOR 2.36; 95% CI: 1.21–4.59). When two or more sentinel organisms were isolated within 5 days, these HO-BSI events were associated with 34% increased odds of mortality (aOR 1.34; 95% CI: 1.17–1.53). HO-BSI caused by AMR isolates had nearly 60% increased odds of mortality (aOR 1.57; 95% CI 1.4–1.77). As compared to medical wards, mortality in surgical wards was lower (aOR 0.34; 95% CI: 0.29–0.4), and mortality in ICU was slightly higher (aOR 1.24; 95% CI: 1.07–1.45). There was no significant association between length of hospitalization before onset of HO-BSI and mortality.

## 4. Discussion

In this study, HO-BSI caused by eight sentinel bacteria occurred in 0.28% of admissions and 0.87% of admissions with LOS greater than three days. Mortality after these HO-BSI events was high: 14-day, 30-day and 1-year mortality were 30.6%, 40.2% and 66.5%, respectively. Over 44% of HO-BSIs with sentinel bacteria were caused by AMR organisms.

In a systematic review by Goto et al. [4], the IR of HO-BSI was 6 per 1000 admissions in the USA; 2.7 per 1000 admissions in France; 3.5 per 1000 admissions in England; 8.4 per 1000 admissions in Spain and 2.7 per 1000 admissions in Finland. Our study included only eight sentinel bacteria; we cannot directly compare the IR to other studies. However, based on unpublished data from the NIARIC on BSI caused by all organisms during 3 months in 2019, we estimated that these eight bacteria account for 70% of all HO-BSIs (not including bacteria classified as skin contaminants [12]). Thus, we estimate that the overall IR of HO-BSI in Israel in adult patients is likely close to 4 per 1000 admissions.

Goto et al. [4] estimated that the CFR for HO-BSIs was between 15–30% in the USA and between 12–32% in Europe. However, most studies included in their analysis reported on in-hospital mortality, which underestimates the true CFR, as some patients die at home or at a post-acute-care facility soon after discharge. Since mortality data in our study came from the national death registry, we avoided this limitation.

In a study by Wisplinghoff et al. [13], the time from hospital admission to infection varied by infecting organism: 13 days for *E. coli*, 16 days for *S. aureus*, 22 days for *Klebsiella* species and 26 days for *Acinetobacter* species. We found similar results: *S. pneumoniae, E. coli* and *S. aureus* BSI were earlier events (median time from admission to HOBSI was 9–12 days), and *K. pneumoniae*, *P. aeruginosa*, *A. baumannii* and *Enterococcus* species BSI were later events (median time from admission to infection was 16–20 days). The high frequency of BSI caused by *E. coli* and *S. aureus*, pathogens common in the community, may also reflect misclassification of these infections as hospital-onset; they may have begun before hospital admission but diagnosed late because of a delay in taking blood cultures. In the case of *E. coli*, BSI early in hospitalization could also reflect translocation of the patient’s own flora following invasive procedures or devices. In contrast, pathogens such as *A. baumannii* and *P. aeruginosa* are nosocomially acquired and thus cause BSI later in the course of hospitalization.

In a study by Leibovici et al. [14], there was a positive linear relationship between the proportion of BSI caused by antibiotic-resistant organisms and the day of BSI onset, with no specific threshold during the first 3 weeks of hospitalization. Our study had similar findings, with increased resistance among all sentinel organisms as day of hospitalization progressed. In a study by Kadri et al. [8], patients with BSI caused by antibiotic-resistant organisms were nine times more likely to receive inappropriate empirical antibiotic treatment than those with BSI caused by antibiotic-susceptible organisms. Inappropriate empirical antibiotic treatment increased the odds of in-hospital mortality by 46%. Similar findings were found by Leibovici et al. [15] and in the ICU setting by Ibrahim et al. [16]. We did not have data on antibiotic treatment, but our finding of nearly 60% higher 30-day mortality following HO-BSI events caused by AMR isolates may reflect, at least in part, delayed or less effective therapy.

Often surveillance systems are confined to selected university or tertiary care hospitals that admit complex cases, which may cause referral bias [5]. Our study encompassed all acute care hospitals in Israel, thus avoiding selection bias, and provided data on nationwide incidence. As expected, incidence was twice as high in large tertiary care hospitals as compared to smaller hospitals (3.4 vs. 1.6 per 1000 admissions), reflecting the difference in case mix. Interestingly, there was no significant difference in 14-day and 30-day mortality by hospital size.

In a population-based cohort study from Denmark [17], mortality following BSI was significantly higher compared to controls without a BSI after adjusting for age and sex: 30-day mortality was 22% vs. 0.2%, 1-year mortality was 41.4% vs. 2.6% and 5-year mortality was 63.0% vs. 16.8%. In a study by Prescott et al. [18], sepsis was associated with a 22% increase in late mortality (>30 days and up to two years), compared with matched non-hospitalized adult controls. Leibovici et al. [19] found that long-term survival of patients was influenced by the severity of the original infection and the appropriateness of treatment. Indeed, we found high 1-year mortality for patients following HO-BSI.

Goto et al. [4] estimated that BSI is among the top seven causes of death in middle- and high-income countries and contributes to more deaths than any other infection. Despite this observation, the subject has received little attention, national data are scarce and there are no mortality estimates from the past decade. Population-based BSI surveillance systems have been recommended for years [5,20], but implementation is lagging. Monitoring HO-BSI is important to assess the impact of specific pathogens and antibiotic resistance, as well as to assess the effectiveness of preventive interventions. Furthermore, in recent decades, most surveillance and infection control attention has focused on preventing device-related infections, primarily central-line-associated bloodstream infections (CLABSI). We found that most HO-BSIs (86%) were acquired outside of the ICU, and 67% of events were acquired in medical wards. Thus, attention should be shifted beyond device-associated BSI, and specific interventions are required outside of the ICU, especially in medical wards.

It is estimated that at least 50% of HO-BSIs are preventable with adequate infection control measures [21]. For instance, in England, the introduction of national guidelines for prevention of HAIs and mandatory surveillance and reporting of methicillin-resistant *S. aureus* (MRSA) BSI in 2001 led to an 80% reduction in MRSA infection rates [22]. Compliance with CLABSI bundles has decreased CLABSI rates by 33% in the USA [23]. Targeted infection control interventions have led to a reduction in ICU CLABSI rates in Israeli hospitals over the last decade from 7.4 to 2.3 events per 1000 central-line days [24]. Furthermore, improving sepsis care, rapid diagnosis and antimicrobial stewardship programs decrease mortality in patients with HO-BSI [25,26].

The strengths of this study include the nationwide sample and the use of a national death registry that enabled accurate tracking of deaths after hospital discharge. Our surveillance system encompasses all Israeli hospitals, of varying bed size and academic affiliations, thus representing a large case mix.

This study had several limitations. The detection of BSI depends on adequate practices such as timing, volume of blood drawn and number of culture sets, as well as physicians’ threshold to order a blood culture [5]. Since our surveillance data included only positive blood cultures, we did not evaluate the total number of blood cultures obtained and diagnostic variation between hospitals. Laboratory surveillance may overestimate HO-BSI rates, as detection of bacteremia in the first few days may reflect delayed detection of community-acquired BSI and not true HO-BSI. As we lacked clinical data, the mortality data presented here represent all-cause mortality and not infection-related mortality. Survival data were not available for all patients; however, we do not think this should bias our estimate of mortality.

In conclusion, this nationwide study highlights the incidence of HO-BSI in Israel from eight sentinel bacteria, the associated high mortality and important role of antibiotic resistance, and can serve to direct efforts and resources for HO-BSI prevention and treatment.

## Figures and Tables

**Table 1:** Characteristics of hospital-onset bloodstream infections (HO-BSIs) caused by sentinel bacteria among adult patients, Israel, 2018–2019.

Characteristics	*N* = 6752	
Female sex, *n* (%)	2874	(42.6)
Age, mean (±SD)	68.5	(±16.3)
Days from admission to bacteremia, median (IQR)	15	(8–29)
Hospital size (beds), *n* (%)		
<300	673	(10)
300–700	2393	(35.4)
>700	3686	(54.6)
Department at onset, *n* (%)		
Medical	4533	(67.1)
ICU	943	(14)
Surgical	1184	(17.5)
Not reported	92	(1.4)
Organism, *n* (%)		
*Escherichia coli*	1491	(19.8)
*Klebsiella pneumoniae*	1659	(22.1)
*Staphylococcus aureus*	1315	(17.5)
*Pseudomonas aeruginosa*	1175	(15.6)
*Enterococcus faecalis*	778	(10.4)
*Enterococcus faecium*	405	(5.4)
*Acinetobacter baumannii*	654	(8.7)
*Streptococcus pneumoniae*	43	(0.6)
Events with ≥2 sentinel organisms isolated within 5 days, *n* (%)	675	(10)
Events with antimicrobial resistant organisms ^a^, *n* (%)	2984	(44.2)

^a^ Defined as resistance to key antimicrobial agents: penicillin resistance in *S. pneumoniae*; oxacillin or cefoxitin resistance in *S. aureus*; vancomycin or teicoplanin resistance in *E. faecalis* and *E. faecium*; extended-spectrum cephalosporin (third and fourth generation) resistance in *E. coli* and *K. pneumonia* and non-susceptibility to imipenem or meropenem in *E. coli*, *K. pneumoniae*, *A. baumannii* and *P. aeruginosa*.

**Table 2:** Incidence rates of hospital-onset bloodstream infections (HO-BSIs) caused by sentinel bacteria among adult patients, Israel, 2018–2019.

Category		*n*	BSI per 1000 Admissions (95% CI)		BSI per 1000 Admissions at Risk ^a^ (95% CI)		BSI per 10,000 Patient-Days (95% CI)		BSI per 10,000 Patient-Days at Risk ^b^ (95% CI)	
All events		6752	2.84	(2.77–2.91)	8.66	(8.45–8.86)	6.88	(6.72–7.05)	9.63	(6.72–7.05)
Hospital day of onset	4–7	1528	1.96	(1.86–2.06)	Not calculated (denominator data not available)		2.18	(2.07–2.29)	Not calculated (denominator data not available)	
	8–14	1793	6.84	(6.53–7.16)			4.06	(3.87–4.25)		
	≥15	3431	35.04	(33.89–36.19)			12.43	(12.01–12.84)		
Hospital size (beds)	<300	673	1.61	(1.49–1.73)	5.66	(5.23-6.08)	4.64	(4.29–4.99)	7.31	(4.29–4.99)
	300–700	2393	2.71	(2.6–2.81)	7.96	(7.64-8.28)	6.61	(6.34–6.87)	9.25	(6.34–6.87)
	>700	3686	3.43	(3.32–3.55)	10.23	(9.9–10.56)	7.78	(7.53–8.03)	10.53	(7.53–8.03)
Organism	*Escherichia coli*	1491	0.63	(0.6–0.66)	1.91	(1.81–2.01)	1.52	(1.44–1.6)	2.13	(1.44–1.6)
	*Klebsiella pneumoniae*	1659	0.70	(0.66–0.73)	2.13	(2.03–2.23)	1.69	(1.61–1.77)	2.37	(1.61–1.77)
	*Staphylococcus aureus*	1315	0.55	(0.52–0.58)	1.69	(1.6–1.78)	1.34	(1.27–1.41)	1.88	(1.27–1.41)
	*Pseudomonas aeruginosa*	1175	0.49	(0.47–0.52)	1.51	(1.42–1.59)	1.20	(1.13–1.27)	1.68	(1.13–1.27)
	*Enterococcus faecalis*	778	0.33	(0.3–0.35)	1.00	(0.93–1.07)	0.79	(0.74–0.85)	1.11	(0.74–0.85)
	*Enterococcus faecium*	405	0.17	(0.15–0.19)	0.52	(0.47–0.57)	0.41	(0.37–0.45)	0.58	(0.37–0.45)
	*Acinetobacter baumannii*	654	0.28	(0.25–0.3)	0.84	(0.77–0.9)	0.67	(0.62–0.72)	0.93	(0.62–0.72)
	*Streptococcus pneumoniae*	43	0.02	(0.01–0.02)	0.06	(0.04–0.07)	0.04	(0.03–0.06)	0.06	(0.03–0.06)
Events with antimicrobial resistant organisms ^c^		2984	1.26	(1.21–1.3)	3.83	(3.69–3.96)	3.04	(2.93–3.15)	4.26	(2.93–3.15)

^a^ Admissions at risk were defined as admissions lasting at least 4 days. ^b^ Patient-days at risk were defined as patient-days counts beginning on hospital day 4. ^c^ Defined as resistance to key antimicrobial agents: penicillin resistance in *S. pneumoniae*; oxacillin or cefoxitin resistance in *S. aureus*; vancomycin or teicoplanin resistance in *E. faecalis* and *E. faecium*; extended-spectrum cephalosporin (3rd and 4th generation) resistance in *E. coli* and *K. pneumonia* and non-susceptibility to imipenem or meropenem in *E. coli*, *K. pneumoniae*, *A. baumannii* and *P. aeruginosa*.

**Table 3:** Distribution of sentinel organisms by week of hospital-onset bloodstream infection (HO-BSI) onset, and antimicrobial resistance (AMR) status ^a^.

Organism	Week 1		Week 2		Week 3		Week 4+	
	% of Total	% AMR	% of Total	% AMR	% of Total	% AMR	% of Total	% AMR
*Escherichia coli*	25.09	41.39	21.68	39.39	21.17	47.77	14.66	50.63
*Klebsiella pneumoniae*	17.17	56.29	22.49	58.88	19.79	67.97	25.74	72.02
*Staphylococcus aureus*	24.79	21.31	18.24	34.35	15.42	51.11	13.33	60.66
*Pseudomonas aeruginosa*	11.46	12.04	13.85	12.41	16.80	14.29	18.98	26.46
*Enterococcus faecalis*	10.62	0	9.55	0.53	9.51	0	11.12	0
*Enterococcus faecium*	2.94	14.29	4.50	20.22	6.68	11.54	6.98	21.69
*Acinetobacter baumannii*	6.84	71.93	9.15	85.08	10.37	86.78	8.79	89.92
*Streptococcus pneumoniae*	1.08	0	0.56	0	0.26	0	0.41	0

^a^ Defined as resistance to key antimicrobial agents: penicillin resistance in *S. pneumoniae*; oxacillin or cefoxitin resistance in *S. aureus*; vancomycin or teicoplanin resistance in *E. faecalis* and *E. faecium*; extended-spectrum cephalosporin (3rd and 4th generation) resistance in *E. coli* and *K. pneumonia* and non-susceptibility to imipenem or meropenem in *E. coli*, *K. pneumoniae*, *A. baumannii* and *P. aeruginosa*.

**Table 4:** Mortality (all cause) following hospital-onset bloodstream infections (HO-BSIs) caused by sentinel bacteria among adult patients, Israel, 2018–2019.

Category		*N* ^a^	14-Day Mortality % (95% CI)		30-Day Mortality % (95% CI)		*N* ^a^	1-Year Mortality % (95% CI)		Adjusted ^b^ OR for Death within 30 Days (95% CI)
All events		5907	30.6	(28.5–32.75)	40.2	(38.18–42.13)	3839	66.5	(64.67–68.33)	
Sex	Male	3337	31.2	(28.35–33.98)	41.2	(38.57–43.78)	2164	66.9	(64.49–69.34)	1.1 (0.99–1.22)
	Female	2543	29.8	(26.55–33.06)	38.7	(35.65–41.74)	1648	65.9	(63.08–68.72)	Reference
Age (years)	18–44	528	14.2	(6.3–22.11)	19.9	(12.25–27.52)	347	39.5	(31.3–47.67)	
	45–64	1385	24.8	(20.27–29.4)	33.3	(28.98–37.59)	879	58.6	(54.34–62.84)	
	65–79	2285	30.7	(27.26–34.09)	39.9	(36.69–43.05)	1489	66.8	(63.83–69.68)	
	≥80	1709	40.3	(36.65–43.98)	52.4	(49.1–55.64)	1124	80.7	(78.13–83.26)	
	Age, as continuous variable									1.03 (1.02–1.03)
Hospital day of onset	4–7	1362	27.4	(22.86–31.91)	35.9	(31.65–40.16)	898	56.7	(52.38–60.99)	Reference
	8–14	1577	31.1	(27.04–35.23)	39.7	(35.86–43.53)	998	65.5	(61.89–69.17)	
	≥15	2968	31.8	(28.87–34.81)	42.4	(39.62–45.08)	1943	71.5	(69.17–73.91)	1.1 (0.99–1.23)
Hospital size (beds)	<300	588	32.3	(25.66–38.96)	42.0	(35.85–48.16)	375	72.3	(66.94–77.6)	Non-significant ^b^
	300–700	1780	30.4	(26.58–34.32)	39.8	(36.17–43.38)	1162	66.7	(63.38–70.01)	
	>700	3539	30.4	(27.68–33.18)	40.0	(37.49–42.59)	2302	65.5	(63.06–67.87)	
Department	Medical	4028	33.8	(31.25–36.28)	44.4	(42.06–46.67)	2672	72.8	(70.85–74.81)	Reference
	ICU	816	36.2	(30.67–41.63)	45.5	(40.4–50.53)	526	64.1	(58.95–69.19)	1.24 (1.07–1.45)
	Surgical	1056	14.6	(9.01–20.16)	20.3	(14.88–25.65)	634	42.6	(36.69–48.48)	0.34 (0.29–0.4)
Organism	*Escherichia coli*	1280	23.3	(18.48–28.08)	32.0	(27.51–36.55)	794	56.2	(51.57–60.78)	Reference
	*Klebsiella pneumoniae*	1403	28.6	(24.16–33)	38.6	(34.53–42.73)	880	68.2	(64.45–71.91)	1.14 (0.96–1.35)
	*Staphylococcus aureus*	1171	30.0	(25.18–34.77)	40.2	(35.79–44.65)	774	65.0	(60.82–69.16)	1.29 (1.08–1.53)
	*Pseudomonas aeruginosa*	1043	31.1	(26.03-36.1)	39.8	(35.08–44.5)	704	67.0	(62.8–71.29)	1.42 (1.18–1.71)
	*Enterococcus faecalis*	695	29.4	(23.1–35.6)	41.0	(35.3–46.72)	450	73.3	(68.56–78.1)	1.42 (1.16–1.75)
	*Enterococcus faecium*	340	38.2	(29.88–46.59)	50.6	(43.12–58.06)	216	74.1	(67.28–80.86)	2.16 (1.66–2.79)
	*Acinetobacter baumannii*	605	55.2	(49.87–60.54)	64.6	(59.89–69.37)	425	83.1	(79.15–86.97)	2.85 (2.3–3.55)
	*Streptococcus pneumoniae*	38	42.1	(17.91–66.3)	50.0	(27.52–72.48)	28	64.3	(42.15–86.42)	2.36 (1.21–4.59)
Events with ≥2 sentinel organisms isolated within 5 days	No	5318	30.0	(27.74–32.24)	39.3	(37.19–41.38)	3453	65.7	(63.76–67.66)	Reference
	Yes	589	36.3	(29.89–42.78)	48.0	(42.23–53.87)	386	73.6	(68.45-78.7)	1.34 (1.17–1.53)
Events with antimicrobial resistant organisms ^c^	No	3338	25.3	(22.35–28.22)	34.1	(31.34–36.85)	2132	59.3	(56.58–62)	Reference
	Yes	2569	37.6	(34.51–40.62)	48.0	(45.25–50.82)	1707	75.5	(73.17–77.86)	1.57 (1.4–1.77)

^a^ The number of events in each category does not add up to the total number of events because of missing data. ^b^ Multivariable model adjusting for age, sex, day of HO-BSI onset, department, organism, events with ≥2 sentinel organisms and events with antimicrobial resistant organisms. Hospital size was non-significant in univariate analysis and therefore was excluded from the model. ^c^ Defined as resistance to key antimicrobial agents: penicillin resistance in *S. pneumoniae*; oxacillin or cefoxitin resistance in *S. aureus*; vancomycin or teicoplanin resistance in *E. faecalis* and *E. faecium*; extended-spectrum cephalosporin (3rd and 4th generation) resistance in *E. coli* and *K. pneumonia* and non-susceptibility to imipenem or meropenem in *E. coli*, *K. pneumoniae*, *A. baumannii* and *P. aeruginosa*.

## Data Availability

The data presented in this study are available on request from the corresponding author.

## Supplementary Materials

The following are available online at https://www.mdpi.com/article/10.3390/microorganisms10051009/s1, Table S1. STROBE Statement.
