# Supplementary material for: Hospital-Onset Bloodstream Infections Caused by Eight Sentinel Bacteria: A Nationwide Study in Israel, 2018–2019
Source: Microorganisms. 2022 May 11;10(5):1009. doi: 10.3390/microorganisms10051009 (PMC9147328; doi:10.3390/microorganisms10051009)
Supplement: Supplementary file 1 [file microorganisms-10-01009-s001.zip › microorganisms-1704045-supplementary.pdf]

Table S1. STROBE Statement.

	Item No.	Recommendation	Where Found in Manuscript
**Title and abstract**	1	(*a*) Indicate the study's design with a commonly used term in the title or the abstract	Methods section of abstract
		(*b*) Provide in the abstract an informative and balanced summary of what was done and what was found	Methods and results sections of abstract
**Introduction**			
Background/rationale	2	Explain the scientific background and rationale for the investigation being reported	Introduction, first to third paragraphs
Objectives	3	State specific objectives, including any prespecified hypotheses	Introduction, fourth paragraph
**Methods**			
Study design	4	Present key elements of study design early in the paper	Methods section
Setting	5	Describe the setting, locations, and relevant dates, including periods of recruitment, exposure, follow-up, and data collection	Methods, Study setting and participants, and Data collection
Participants	6	(*a*) *Cohort study*—Give the eligibility criteria, and the sources and methods of selection of participants. Describe methods of follow-up<br>*Case-control study*—Give the eligibility criteria, and the sources and methods of case ascertainment and control selection. Give the rationale for the choice of cases and controls<br>*Cross-sectional study*—Give the eligibility criteria, and the sources and methods of selection of participants	Methods, Data collection and Laboratory methods
		(*b*) *Cohort study*—For matched studies, give matching criteria and number of exposed and unexposed	NA

		Case-control study—For matched studies, give matching criteria and the number of controls per case	
Variables	7	Clearly define all outcomes, exposures, predictors, potential confounders, and effect modifiers. Give diagnostic criteria, if applicable	Methods, Definitions
Data sources/ measurement	8	For each variable of interest, give sources of data and details of methods of assessment (measurement). Describe comparability of assessment methods if there is more than one group	Methods, Definitions
Bias	9	Describe any efforts to address potential sources of bias	NA
Study size	10	Explain how the study size was arrived at	NA
Quantitative variables	11	Explain how quantitative variables were handled in the analyses. If applicable, describe which groupings were chosen and why	NA
Statistical methods	12	(*a*) Describe all statistical methods, including those used to control for confounding	Methods, Incidence, Mortality and Statistical analysis
		(*b*) Describe any methods used to examine subgroups and interactions	Methods, Statistical analysis
		(*c*) Explain how missing data were addressed	NA
		(*d*) *Cohort study*—If applicable, explain how loss to follow-up was addressed<br>*Case-control study*—If applicable, explain how matching of cases and controls was addressed<br>*Cross-sectional study*—If applicable, describe analytical methods taking account of sampling strategy	NA
		(*e*) Describe any sensitivity analyses	NA
**Results**			

Participants	13	(a) Report numbers of individuals at each stage of study—eg numbers potentially eligible, examined for eligibility, confirmed eligible, included in the study, completing follow-up, and analysed	Results, first paragraph,
		(b) Give reasons for non-participation at each stage	Results, "Data on mortality"
		(c) Consider use of a flow diagram	NA
Descriptive data	14	(a) Give characteristics of study participants (eg demographic, clinical, social) and information on exposures and potential confounders	Table 1
		(b) Indicate number of participants with missing data for each variable of interest	See 13
		(c) *Cohort study*—Summarise follow-up time (eg, average and total amount)	NA
Outcome data	15	*Cohort study*—Report numbers of outcome events or summary measures over time	Tables 2 & 3
		Case-control study—Report numbers in each exposure category, or summary measures of exposure	
		Cross-sectional study—Report numbers of outcome events or summary measures	
Main results	16	(*a*) Give unadjusted estimates and, if applicable, confounder-adjusted estimates and their precision (eg, 95% confidence interval). Make clear which confounders were adjusted for and why they were included	Tables 2 & 3
		(*b*) Report category boundaries when continuous variables were categorized	NA
		(*c*) If relevant, consider translating estimates of relative risk into absolute risk for a meaningful time period	NA
Other analyses	17	Report other analyses done—eg analyses of subgroups and interactions, and sensitivity analyses	NA
**Discussion**			
Key results	18	Summarise key results with reference to study objectives	Discussion, first paragraph
Limitations	19	Discuss limitations of the study, taking into account sources of potential bias or imprecision. Discuss both direction and magnitude of any potential bias	Discussion, "Some limitations should be discussed"
Interpretation	20	Give a cautious overall interpretation of results considering objectives, limitations, multiplicity of analyses, results from similar studies, and other relevant evidence	Discussion

Generalisability	21	Discuss the generalisability (external validity) of the study results	Discussion, 2nd and 3rd paragraphs
**Other information**			
Funding	22	Give the source of funding and the role of the funders for the present study and, if applicable, for the original study on which the present article is based	Funding
